# Runoff Response to Soil Moisture and Micro-topographic Structure on the Plot Scale

**DOI:** 10.1038/s41598-019-39409-6

**Published:** 2019-02-22

**Authors:** Jiakai Liu, Bernard A. Engel, Yu Wang, Yanan Wu, Zhenming Zhang, Mingxiang Zhang

**Affiliations:** 1 Beijing Forestry University, School of Nature Conservation, Beijing, CN China; 2 Purdue University, Agricultural and Biological Engineering, W. Laffayette, IN USA

## Abstract

Structural hydrological connectivity has been proposed to describe the geological structure of the landscape as well as to explain hydrological behaviors. Indices based on the topological or soil condition were developed to interpret their relationships. While previous studies mainly focused on well-instrumented catchments which are narrow in humidity or temperate zone, the hydrological responses to structural connectivity at the plot and hill slope scale as well as in arid or semi-arid climate conditions remain unclear. This study was conducted in the semi-arid mountainous region of northern China in Haihe Basin which is the source of water of about 350 million people. Experiments were conducted during the rainy season in 2012 and 2013 using four runoff plots. Two indices, flow path length (FL) based on topography and integral connectivity scale length (ICSL) based on soil moisture conditions, developed to represent hydrological connectivity structure and the runoff response to rainfall were analyzed. The results showed that the surface runoff coefficient was strongly and positively linearly correlated to FL, and the correlation between subsurface flow and ICSLs was quadratic. Plots with shorter FL required more rainfall to generate surface runoff. In the shallow soil layer, when the ICSLs are relatively low, the soil can store more water and less rainfall feeds subsurface runoff. Further analysis indicated that improved shallow soil connectivity conditions might enhance the water-holding capacity and lead to lower water yields for each event. This study demonstrated that hydrological structure connectivity could explain the mechanism of runoff generation in semi-arid areas while further experiments should be undertaken to find the threshold-like relationship between FL and surface runoff as well as the influence of plant cover on hydrological behaviors.

## Introduction

In recent years, the concept of hydrological connectivity has been proposed and studied to describe the geological/ecological structure of the landscape as well as to explain both hydrological behavior and the relationship between hydrological and ecological processes^1–8^. Despite different concepts of this new term emanating as a function of the different types of environments studied and research scales used^9^, the common view is that the configuration of geological units (including vegetation, soil moisture, and topographical characteristics), along with climatic conditions, influence the processes of water-mediated transport of matter, energy, and organisms^10,11^. This configuration is considered static or structural hydrological connectivity, while the processes denote the dynamic or functional components^9^.

Static or structural hydrological connectivity refers to the spatial pattern of hydrological response units^12^ and has been studied for decades^13–16^. Some indices have been well developed for this purpose^9^ and the most commonly used methods include semivariograms^17,18^, entropy^13,17^, binarization by threshold^9,17^, flow path length (FL)^16^, and integral connectivity scale length (ICSL)^13,17,18^. Ali *et al*. (2010) tested almost all of these structure metrics in a small catchment and found that only a few metrics were significantly correlated with both meteorological conditions and hydrological responses at the outlet. Mayor *et al*. (2008) proposed the use of the FL method for capturing the connectivity structure at both the plot and watershed scales and demonstrated that the surface runoff response could be well explained by this index. ICSL based on soil moisture data has been reported in an extremely limited number of references^13,17,19,20^ because of the complexity in its measurement and calculation. It has, however, been recommended as an efficient method for capturing hydrological structure patterns^9,13,20^, especially soil moisture conditions^17^, which play an important role in runoff generation^21–23^.

Most previous research on structural connectivity has looked at the relationship between connectivity and hydrological behavior as well as ecological processes^13,15–17,24–26^. Most research focused on runoff generation as a function of structural connectivity has used model simulations^17,20^. Other studies focused on functional hydrological connectivity used the “breakthrough volume” theory by measuring the subsurface water table/soil moisture and analyzing the relationship between precipitation, catchment geology, and change in flow at the outlet^1,8,27–29^. Few studies tried to link structural connectivity with hydrological data at the plot scale, which is meaningful for understanding both hydrological behavior as well as its relationship with other ecological processes such as community succession. In addition, previous case studies only concentrated on a few instrumented experimental catchments which were narrow in humidity or temperate zone. Thus, studies of the processes across different climatic conditions, especially in arid or semiarid areas, is a next step to gain a better understanding of hydrological connectivity. The mountainous region in Haihe Basin in northern China is the source of water for many cities including Beijing. Hydrological studies here are not only meaningful scientifically but also important for the water supply of about 350 million people^30^.

The aims of this work were three-fold: (1) collecting hydrological data at the study site, (2) measuring the hydrological connectivity structure based on soil moisture conditions and topographic metrics, and (3) linking the relationship between runoff generation and structural hydrological connectivity to obtain a better understanding of hydrological processes in the semi-arid mountainous areas in north China.

## Results

### Antecedent soil moisture pattern and runoff events

**Figure 1:**
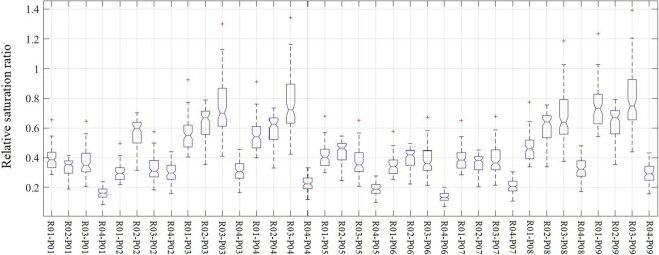
Antecedent soil moisture conditions of each runoff plot before each runoff generation event. R01–R04 denote the runoff plots and P01–P09 refer to the precipitation events.

**Figure 2:**
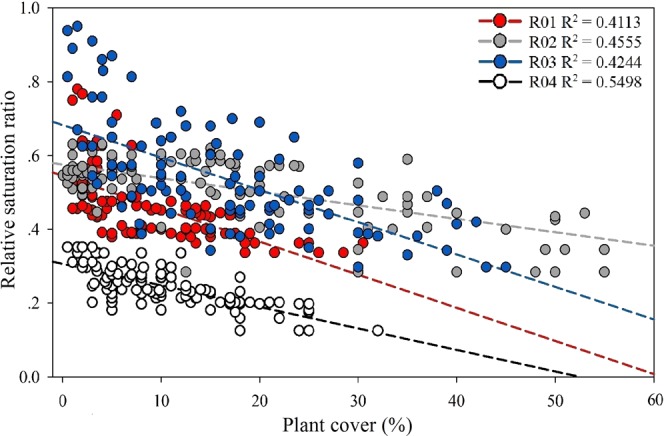
Relationship between plant cover and relative saturation ratio.

The antecedent soil moisture conditions of each event in different runoff plots are shown in Fig. 1. The relative saturation ratio (RSR) in R04 (0.24 ± 0.07) was significantly smaller than that of the other three plots (p < 0.001) with values of 0.47 ± 0.13, 0.51 ± 0.12, and 0.54 ± 0.20 (p < 0.05) for R01, R02, and R03, respectively. This difference might be due to the plant pattern. In R04, there were 24 trees, while in the other three plots there were only 18. Further, the diameter at breast height (DBH) in plot 4 (22.6 ± 3.4 cm) was significantly larger than the average DBHs of the other plots (17.6 ± 2.6 cm, p < 0.001). The RSR was negatively correlated to the plant cover ratio (Fig. 2). Plants might decrease the RSR by increasing the maximum soil volumetric water content without causing a drier soil condition. Plant roots could lead to a higher total porosity^31–33^ and subsequently more space to store water^34^. The RSR variation also leads to differences of ICSL among different plots.

**Figure 3:**
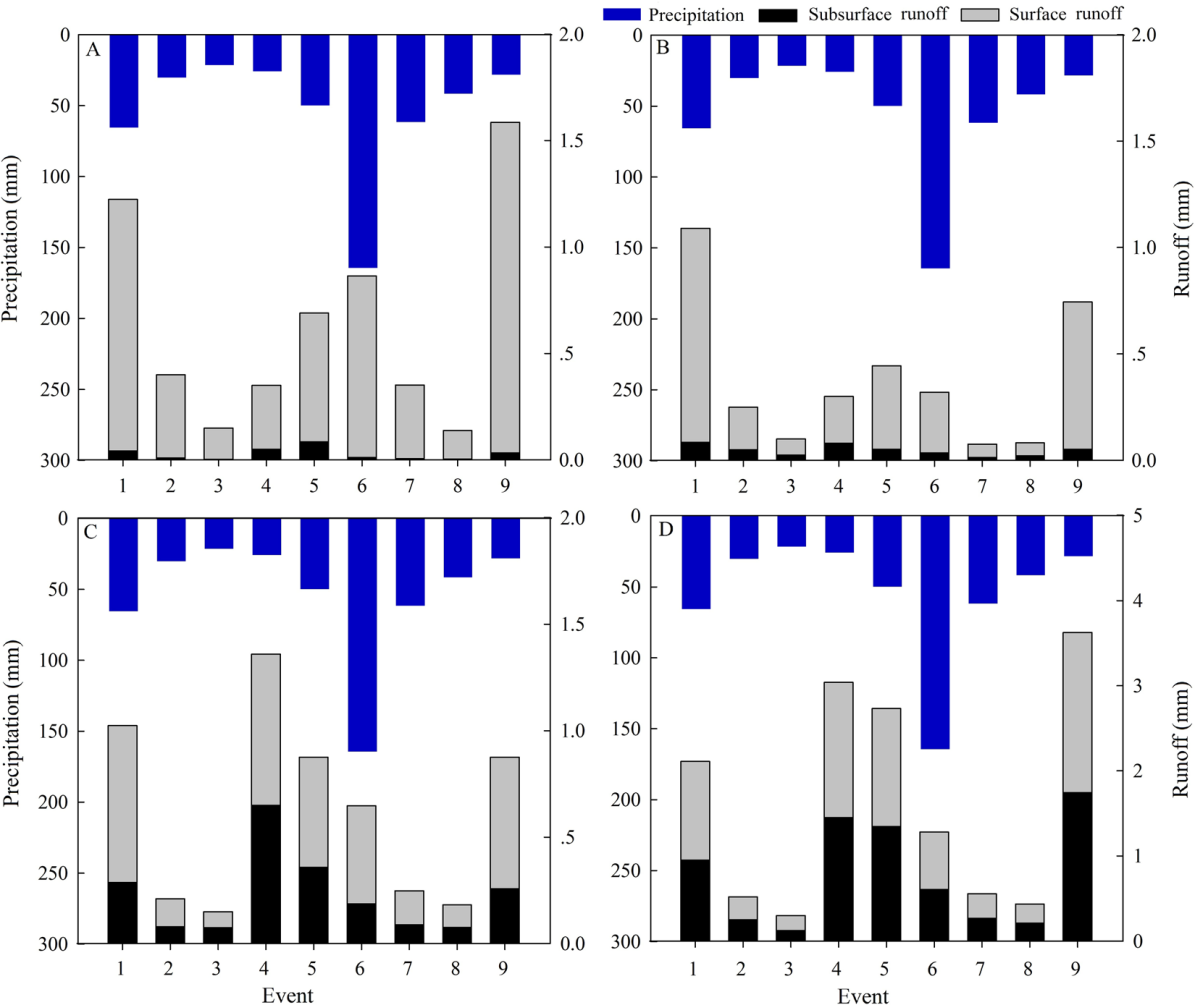
Precipitation and runoff for each runoff generation event. (**A**–**D**) refer to R01, R02, R03, and R04, respectively.

There were nine runoff generation events recorded during the experimental period; their details are shown in Fig. 3. The average shallow subsurface runoff in the four runoff plots was 0.027 mm, 0.046 mm, 0.229 mm, and 0.772 mm, respectively, while the surface runoff was 0.586 mm, 0.286 mm, 0.161 mm, and 0.077 mm, respectively. The total runoff coefficients were not significantly different; their values were 0.015 ± 0.016, 0.008 ± 0.007, 0.025 ± 0.023, and 0.014 ± 0.046 for R01 to R04, respectively. However, the SRC of R01 (0.014 ± 0.015) was larger than that of R03 (0.003 ± 0.004, p = 0.02) and R04 (0.002 ± 0.002, p = 0.03). The SSFC of R04 (0.023 ± 0.024) was significantly higher than the others (p < 0.001).

### FL and surface runoff

**Figure 4:**
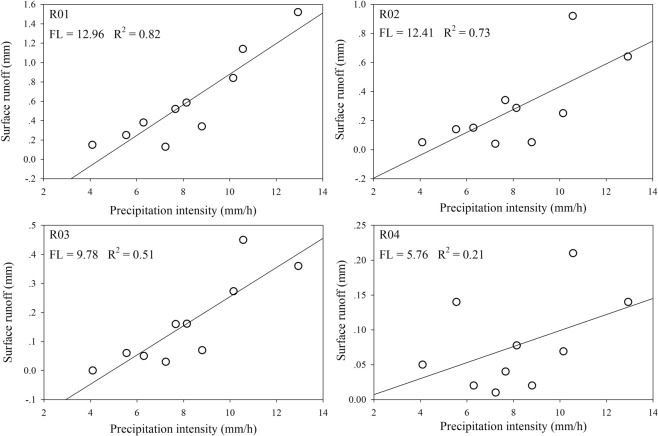
Relationship between precipitation intensity and runoff in different plots. FL: flow path length (m).

The relationship between surface runoff and precipitation intensity of each event are shown in Fig. 4. The FLs of R01 to R04 were 12.96 m, 12.41 m, 9.78 m, and 5.76 m, while the average surface runoff coefficients were 0.013, 0.006, 0.003 and 0.002, respectively. Infiltration and geological condition were considered the two main factors influencing runoff. In the current study, the final infiltration rates of the 4 plots differ from each other insignificantly (Table 1), and thus the topographic condition measured by FL is the main factor which influenced surface runoff. The linear relationship between surface runoff and precipitation is more significant statistically in the plots with higher FL. Both of the regression coefficients and surface runoff coefficients increase as FL increases. FL can be considered a parameter representing surface resistance^35^. Plots with longer FL have less resistance for surface runoff while when plots are too short, more water volume is required to “break through” the resistance from the topographic condition. While limited by the total number of research plots in the current study, a threshold-like relationship^9,36^ between precipitation and surface runoff was not observed.

**Table 1:** Details of the four runoff plots.

Runoff plot	R01	R02	R03	R04
Dominant species	*Platycladus orientalis*	*Pinus tabuliformis*	*Pinus tabuliformis*	*Quercus variabilis*
Elevation (m)	145 m	190 m	430 m	395 m
Aspect (°)	SE 48°	SE 45°	SN 85°	NE 10°
Soil depth (cm)	32 cm	45 cm	35 cm	40 cm
NT (stems)	17	19	18	22
CA (m^2^)	2.19 ± 0.48	7.21 ± 4.12	2.34 ± 0.74	6.96 ± 5.43
DBH (cm)	11.8 ± 4.15	13.8 ± 4.68	10.3 ± 5.25	12.5 ± 4.02
H (m)	6.1 ± 1.8	7.9 ± 3.3	5.8 ± 1.5	7.3 ± 3.3
LAI	3.03	3.55	3.21	4.43

NT: number of trees, CA: mean canopy area, DBH: mean diameter at breath height, H: mean tree height, LAI: leaf area index.

### ICSL and subsurface runoff

**Figure 5:**
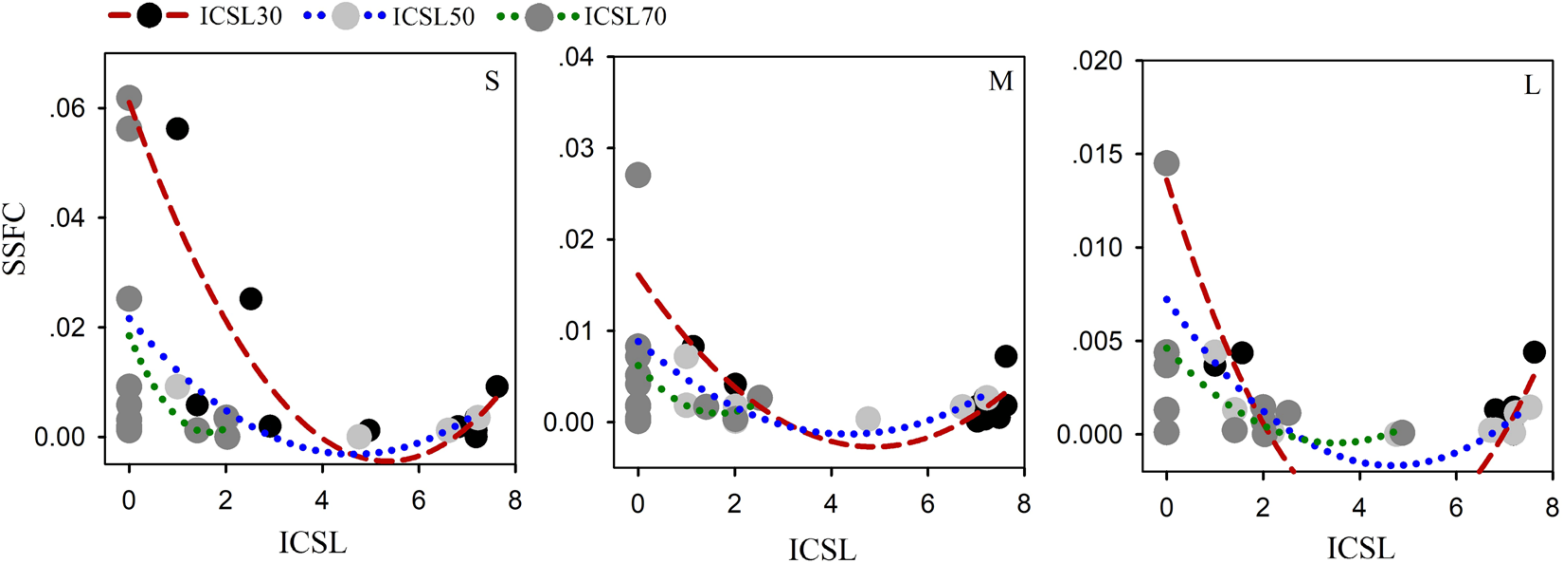
ICSLs of different runoff conditions using different thresholds. ICSL3, ICSL5, and ICSL7 refer to threshold values of 0.3, 0.5, and 0.7, respectively.

The ICSLs were estimated for three thresholds (Fig. 5). For the threshold of 0.3, the average ICSL of R04 (1.98 ± 2.11) was significantly smaller than those of the other plots (p < 0.001). The values of R01, R02, and R03 were similar and they were 6.10 ± 2.02 m, 6.87 ± 0.87 m, and 6.86 ± 1.63 m, respectively. The ICSLs of R04 decreased to 0 for a threshold value of 0.5, which indicated that the relative saturation index of each cell was less than 0.5 for this plot. The ICSLs of R01 to R03 were 2.75 ± 2.15 m, 3.81 ± 3.16 m, and 4.12 ± 3.02 m, respectively. For the threshold value of 0.7, the ICSLs of R01 to R03 were 1.29 ± 1.48 m, 1.16 ± 1.25 m, and 1.13 ± 1.35 m, respectively. The ICSLs decreased as the threshold value increased because the connected path areas become smaller under the higher threshold.

**Figure 6:**
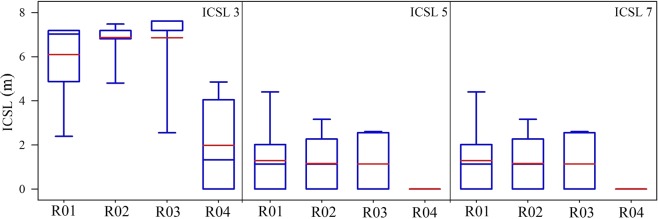
Relationship between ICSL and SSFC for all events in the four runoff plots. S, M, and L refer to the ICSLs in the small, medium and large rainfall events, respectively. ICSL3, ICSL5, and ICSL7 refer to threshold values of 0.3, 0.5, and 0.7, respectively.

The runoff generation events were divided into three categories according to daily rainfall depth: small (rainfall <30 mm/d), medium (30 mm/d < rainfall <60 mm/d), and large (rainfall > 60 mm/d). Regression analyses between ICSLs and SSFCs in the different categories were undertaken. As shown in Fig. 6, the correlation between SSFCs and ICSLs was quadratic. The fits under different thresholds were compared based on the R^2^ values. The R^2^ value of the 0.3 threshold group (0.69 ± 0.20) was higher than that of the 0.5 (0.34 ± 0.18, p = 0.003) and 0.7 (0.18 ± 0.12, p < 0.001) threshold groups. This result indicated that the threshold of the relative saturation index should be about 0.3, and areas with an index higher than 0.3 should be considered connected patches in this semi-arid area. In other words, subsurface flow could break through those girds where the soil volumetric water content was higher than the 30% maximum value. Moreover, for the threshold value of 0.3, the R^2^ value of the average ICSL (0.62 ± 0.24) was smaller than that for the connection ICSL (0.75 ± 0.17); however, the difference is not significant (p = 0.51).

**Figure 7:**
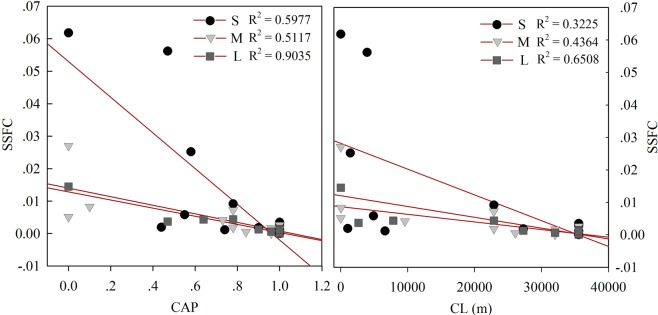
Relationship between SSFC and CAP/CL. S, M, and L refer to the small, medium, and large daily rainfall depths, respectively.

The ICSL values represented the average distance of all connected cell pairs^20^, and according to the fitting curves, under a threshold value of 0.3, the SSFC decreased as the ICSL increased when the ICSLs were less than about 5 m. They did, however, become positively correlated to each other when the ICSLs were higher than 5 m. We propose that in the shallow soil layer, when the ICSLs are relatively small, the soil can restore more water (a better water holding capacity), and less rainfall feeds subsurface runoff. Further, the shallow soil layer provides a larger area with relative saturation above the threshold and subsequently larger ICSLs. CAP and CL were also calculated to verify this hypothesis (Fig. 7). The results show that both the CAP and CL were negatively correlated to SSFC for the three rainfall categories. Thus, larger connected areas, as well as longer total distances of paired cells, would lead to a smaller SSFC. These results support our hypothesis that for the shallow subsurface layer, good structural connectivity enhances infiltration and water-holding ability and, subsequently, decreases water yield^32^.

## Discussion

### Soil moisture and subsurface runoff

ICSLs are based on soil moisture which is considered an important factor influencing subsurface runoff. However, the relationship remains unclear, and results of previous reports are not consistent. A study in the Tarrawarra catchment of southeastern Australia found that ICSLs were strongly correlated to both peak and total discharge^20^. Another study conducted in the Mont Saint-Hillarie catchment in Canada showed no relationship between ICSLs and discharge^13^. The differences may come from climatic conditions, spatiotemporal resolutions as well as plant cover conditions. First, the climatic conditions are the key control for both the mechanism and pattern of runoff^11^. At Tarrawarra, the runoff was dominated by saturation excess overland flow (SOF)^20^ and the response at Mont Saint-Hillarie was controlled by perched water tables^13,37^. HOF, however, dominates in semi-arid regions^31,32^ such as the semi-arid area studied in the current research. Larger ICSLs indicated a better infiltration capacity and, hence, decreased the shallow subsurface runoff^32^. In addition, different scales lead to different hydrological responses^36,38^. Studies focused on the catchment scale calculated ICSLs with different spatial-temporal resolution, while this spatially correlated calculation was probably a source of uncertainty. In the current study, both the runoff coefficient and the structural connectivity were estimated in runoff plots with a 1 m × 1 m spatial resolution. Thus, the spatial heterogeneity could be reflected in the structural connectivity metrics and, subsequently, eliminate the uncertainty. Finally, the impact of vegetation is significant^36^, but has largely been ignored in catchment-scale studies. Plant patterns can change the soil physical characteristics and, subsequently, influence the runoff generation process^33,36,39,40^. In the current study, the threshold was set by the relative saturation index and in plots with different dominant species, which takes into consideration the influence of vegetation pattern (Fig. 2). Thus, the metrics calculated in this research described the connectivity structure more objectively and reasonably.

**Figure 8:**
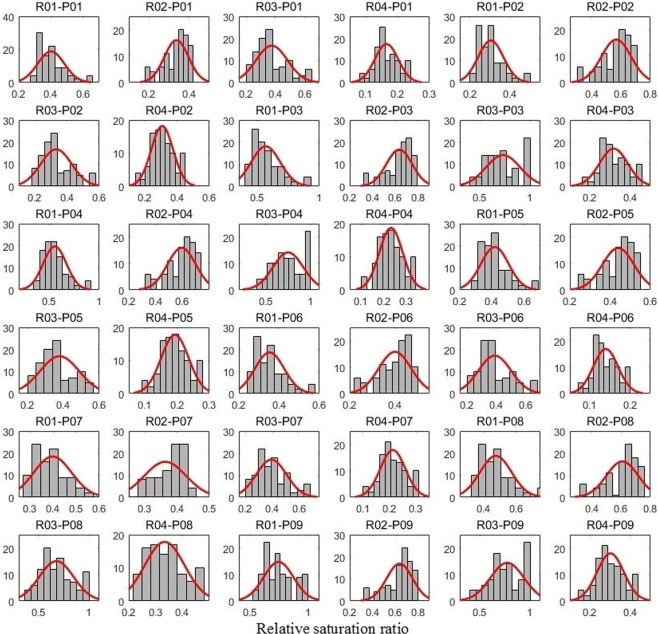
The relative saturation ratio of experiment plots before each rainfall-runoff event. Four plots ware denoted by R01, R02, R03 and R04 while the events are labeled P01 to P09.

In addition, the HOF hypothesis requires a relatively lower infiltration capacity caused by relatively higher soil moisture^41,42^. The traditional way is to set a relatively higher volumetric water content of soil as the threshold, but it does not consider other characteristics of the soil, such as bulk density, which can influence the flow process as well as the infiltration capacity^32,33,40,43,44^. Another question here is how much is “relatively higher”? To improve the previous method, the threshold in this study was set as a proportion (30%, 50% and 70% based on the data) of the maximum soil water volume (Fig. 8), and cells above this moisture content value were considered connected cells. The ICSL values in the current study were also calculated in this way, and we expected a positive linear correlation between ICSL and SSFC, while the analysis gave us a different result. This unexpected result suggests that there might be a dynamic threshold because the rainfall intensity influenced the HOF and, as such, s other methods should be developed to determine this threshold.

### Micro-topography and surface runoff

The surface runoff was analyzed with the FL metric based on micro-topography. This method was proposed by Mayor *et al*. (2008) in a study conducted in a catchment in southeastern Spain. In the current study, the flow direction was determined by the elevation gradient instead of elevation differences, and our results were consist with the previous study that the surface runoff coefficient (R^2^ = 0.66 in the current study and R^2^ = 0.68 in Mayor *et al*.) was strongly and positively linearly correlated to FL.

Mayor *et al*. (2008) also proposed a modified FL that considered the effects of vegetation. They simply treated the vegetation as a sink and marked all the cells with plants as 0. In this way, the total runoff was positively correlated to FL (R^2^ = 0.59); however, the correlation was not significant. Vegetation did seem to influence surface flow, but the method used was oversimplified. Several studies have proven that afforestation would lead to a decrease in annual runoff at the catchment scale^45–47^; however, no evidence has shown that plant areas can not generate runoff. At the plot scale, the role of vegetation characteristics on runoff generation is still controversial. Another study conducted in a mountainous area in Germany claimed that plants played a minor role, while soil and topography characteristics were the key factors^23^, and the existence of roots in the 0~20 cm soil layer would lead to an increase in soil moisture^48^. Another experiment conducted in European vineyards found that the most important factors influencing soil erosion and runoff were vegetation cover and soil moisture^49^. Slope steepness may help to explain this difference. The former study dealt with the hydrological behavior in a mountainous area on a steep slope, while the slope in the latter study was only about 0°~3°. In the current study, the topographical conditions were more similar to the former one. In addition, both the stem density and plant cover did not show any correlation to the surface runoff. Thus, the influence of plants on surface flow was largely ignored in our study.

Uncertainty exists in the current and previous studies. The number of plots was too small to check if there is a threshold-like relationship between FL and surface runoff which was assumed by some studies (Bracken *et al*., 2013; Bachmair and Weiler, 2014a).

### Suggestions for future studies

The structural connectivity itself can be further improved. The physical meaning of ICSL in this study was “the average distance over which pixels are connected”^20^. However, the results of the integral equation (Eq. (7)) represented only the average projected distance of the connected pixels, while structure metrics based on topography reflected changes better^13,15^. Furthermore, the algorithm was complicated, and only a few studies in the literature have applied this metric, which can capture the soil moisture pattern well^13^. Thus, a simplified ICSL might solve both issues. After mapping the connected patches, the connected map could be overlain with the DEM and generate a new raster graphic that has the same resolution as the DEM. The Euclidean distance of each connected paired pixel could then be calculated as well as the number of connected pairs. In this simplified way, the real average distances could be calculated. Furthermore, the threshold value chosen should not be limited by the volumetric soil moisture content. While our research used the relative saturation index based on HOF theory, other parameters, such as hydraulic conductivity, might be more reasonable^41,42,50,51^. However, the methods mentioned above should be tested and verified in future research initiatives, which are beyond the scope of the current study. For FL, the influence of vegetation was ignored in this study, and we suggest the same approach for future studies in similar environments. However, in flat areas such as prairies, bogs, and fens, vegetation may be important. The main priority should focus on how the influence of vegetation characteristics and surface topography can be combined when structural hydrological connectivity in such environments is studied. Furthermore, most of the research on hydrological connectivity only focused on the horizontal direction ignoring the vertical one. In the vertical direction, hydrological behavior in the soil-plant-atmosphere system (SPAS), including precipitation, infiltration, and evapotranspiration, are also important for the water cycle and several other ecological processes^52^. The interaction and connectivity of surface and subsurface flow was also ignored in this study; however, it likely has an effect on hydrological behavior^28,35,53^.

Moreover, structural hydrological connectivity provides us with a new perspective to understand geological/ecological structures^33,54^. Studies should pay more attention to the relationship between hydrological connectivity and other ecological functions^55^ such as nutrient transmission^56^, sediment yield^57,58^, and the distribution of species^5^. For example, sedimentation generation was not discussed in this study; however, it would be meaningful in water and soil conservation efforts and even landslide prevention if a relationship between erosion and hydrological process could be established. Only in this way can we apply the concept of hydrological connectivity to the restoration of ecosystems, the management of water resources, or the preservation of habitat of endangered species. This is far more important and meaningful than studying the hydrological connectivity itself.

## Conclusions

This work measured two structural hydrological connectivity indices based on the soil moisture conditions and topography in the mountainous area of northern China. Surface runoff generation showed a significant positive linger relationship with precipitation in plots with longer FL, and surface runoff coefficient was positively correlated to FL. Meanwhile, the ICSL did not show a specific relationship to SSFC. Further analyses showed that better shallow soil connectivity conditions might enhance infiltration and water-holding capacity. The results of this study are potentially useful in water resource management as water generation can be adjusted by modifying the structural hydrological connectivity. There are many factors that could influence connectivity at the plot scale including micro-topography and forest structure. While the results of this work revealed the linkage between structural connectivity and water yield processes at the plot scale, some of the results could be used to inform water and soil retention projects in areas similar to the study area. However, further studies are required to understand its applicability at larger spatial scales.

## Materials and Methods

### Experimental site

**Figure 9:**
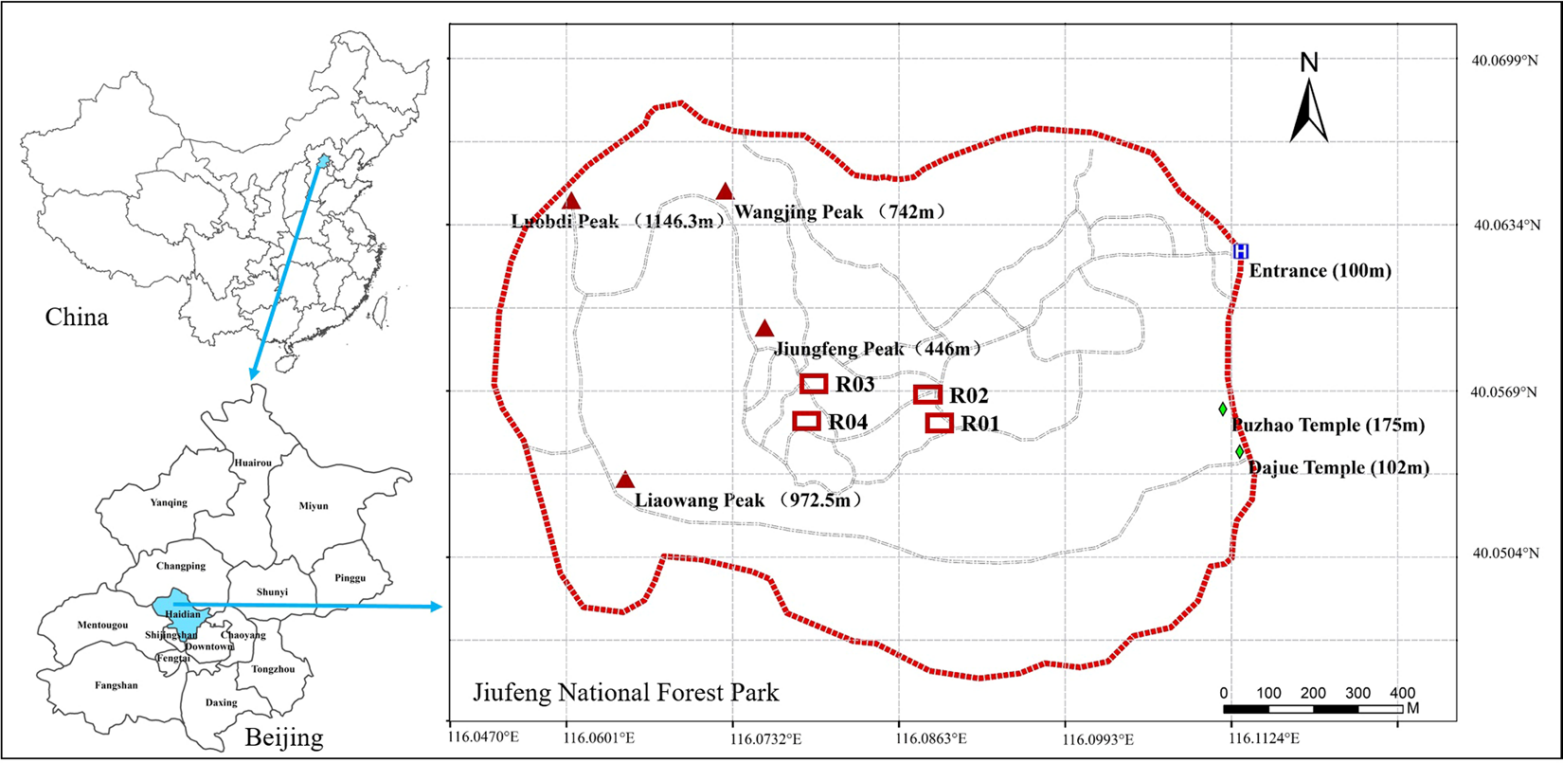
Location of the experimental site and runoff plots. (This map was created using ArcGIS 9.3 and the data resource can be published in the OA journal).

This study was conducted in four instrumented runoff plots built in the Jiufeng National Forest Park (116°09′E, 40°06′N) located in the mountainous area of northern China (Fig. 9) with a semi-arid monsoon climate. The average annual precipitation is about 600 mm, and about 80% of precipitation is concentrated in the period from June to September (China Meteorological Data Sharing Service System http://cdc.cma.gov.cn/). The runoff plots were all 20 m × 5 m with three dominant species which are also the dominant tree species. More details can be found in Table 1. To determine the soil/plant spatial pattern and micro-topographic data, each plot was divided into 100 1 m × 1 m cells. The experiment occurred during the rainy seasons (from June to September) in 2012 and 2013.

### Antecedent soil moisture conditions and plant patterns

Prior to the rainy season in 2012, the maximum soil volumetric water content in the season for the entire plot and the soil’s relative elevation were measured in each cell of all plots. The plant surveys were conducted in every plot at the same time, and the surface cover ratio of the plants in each cell was recorded. During the experimental time period, the soil volumetric water content was recorded at 20 cm depths every 5 days by a time domain reflectometry (TDR)-based potential soil moisture measuring instrument (TRIME-PICO TDR, IMKO Co. Ltd, Germany) for each 1 m × 1 m grid.

Antecedent moisture condition (AMC) and plant distribution can influence hydrological processes^8,59,60^ as well as the hydrological connectivity structure^5,57^. Multiple methods to measure AMCs were introduced in the literature reflecting the different aims of the studies^14,61^. In the current study, the soil relative saturation index was used, which can be expressed as

1 $$RSI=\frac{WV}{W{V}_{max}}\times 100 \% $$

where *RSI* is the relative saturation index, *WV* is the soil volumetric water content of each cell, and *WV*_*max*_ is the maximum soil volumetric water content (the soil volumetric under the saturation condition) of each cell.

### 2.3 Hydrological measurements

Precipitation was recorded for each event using a RG3-M self-recording rain gauge (SEBA Hydrometrie, Kaufbeuren Co. Ltd, Germany). The surface and shallow subsurface runoff from each plot was collected by 2 tanks (2 m × 1 m × 1 m) and measured after each precipitation event. The runoff coefficient of the four runoff plots for each runoff generation event was calculated as

2 $$RC=R/PRE$$

where *PRE* refers to the precipitation. For the shallow subsurface runoff coefficient (SSFC), *R* is the shallow subsurface runoff; for the surface runoff coefficient (SRC), *R* is the subsurface runoff; and for the total runoff coefficient, *R* is the total runoff.

### Hydrological connectivity

Two indicators, FL^16^ and ICSL^13,17^, were used to represent the structural hydrological connectivity^9,11^. Runoff generation was considered as the functional connectivity^1^.

**Figure 10:**
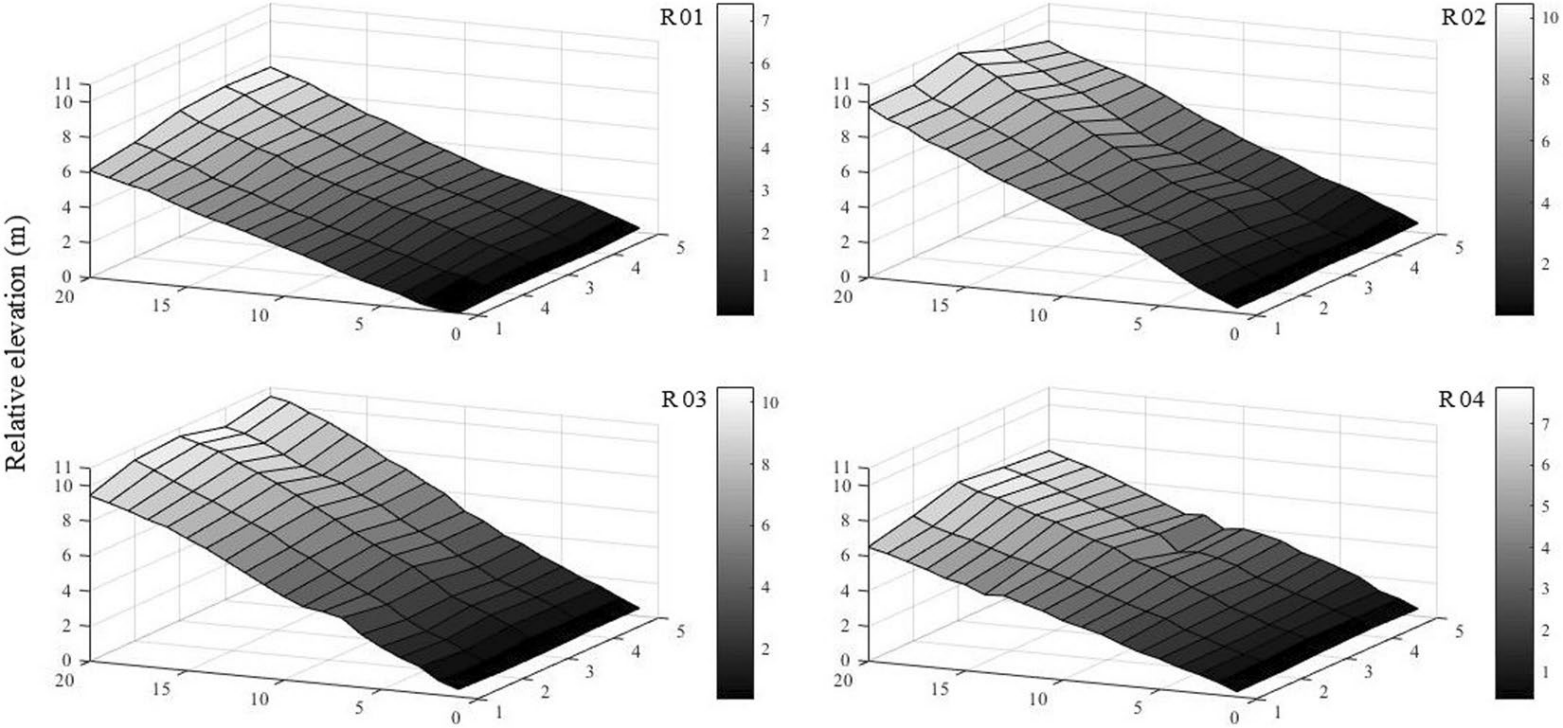
Micro-topography of the experimental plots.

**Figure 11:**
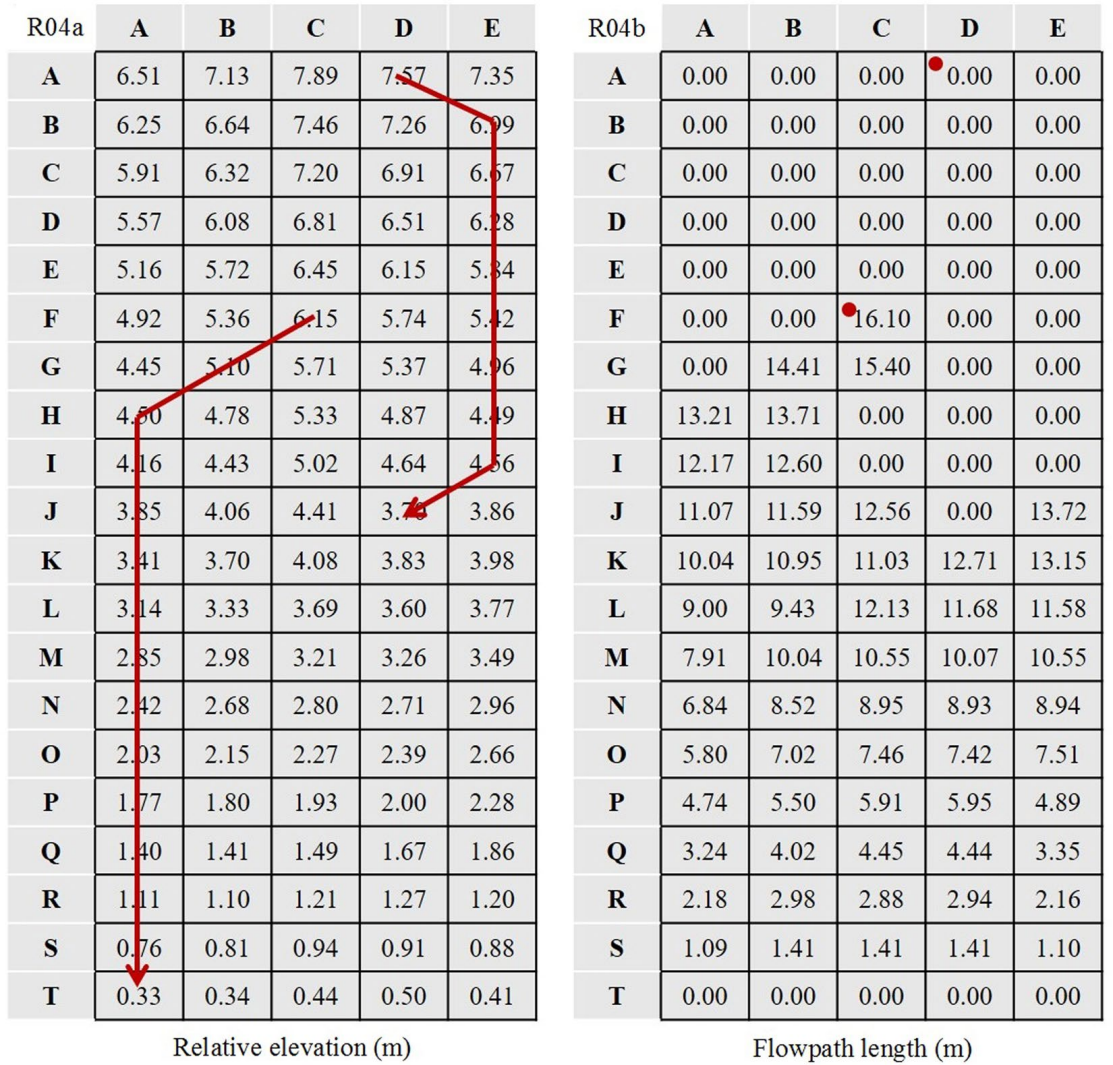
Relative elevation and flowpath length of each cell in R04.

As shown in Fig. 10, we considered the lowest point of each runoff plot as the zero elevation point and measured the relative elevation of each cell. FL is the Euclidean distance of the potential flow path based on the micro-topography of the experimental plots. Mayer *et al*. (2008) first proposed this method to measure the structural connectivity at both plot and catchment scales. A modified FL method was used to represent the surface structural connectivity and was based on the 8-connected pattern^20^. For example, the flow direction in runoff plot R4 was determined according to the elevation gradient (Fig. 3 R04a) calculated as

3 $$ER=\frac{{E}_{ij}-{E}_{rc}}{\sqrt{{({x}_{ij}-{x}_{rc})}^{2}+{({y}_{ij}-{y}_{rc})}^{2}}}$$

where *ER* is the elevation gradient of cell *C*_*ij*_ and one of its connected cells *C*_*rc*_; *E*_*ij*_ and *E*_*rc*_ are the relative elevations of cells *C*_*ij*_ and *C*_*rc*_, respectively; and (*x*_*ij*_, *y*_*ij*_) and (*x*_*rc*_, *y*_*rc*_) are the coordinates of cells *C*_*ij*_ and *C*_*rc*_, respectively. The flow direction was from *C*_*ij*_ to a connected cell with the maximum *ER*. Two examples of *C*_*AD*_ and *C*_*FC*_ are shown in Fig. 11 R04a. Here we assumed that the surface runoff could be collected at the bottom of the plot. Thus, the FL is the Euclidean distance from the start cell to the end cell^16^ when the flow path reached the bottom of the runoff plot; otherwise, the length was considered zero. The FL of each cell in the R04 plot is given in Fig. 11 R04b, and the average FL of each plot was used in further analysis with SRCs. The FLs in this study were calculated in the MATLAB environment. More details can be found in Mayor *et al*. (2008).

The ICSL is the Euclidean distance used to represent the shallow subsurface hydrological structure based on soil moisture conditions^13,17,19,20^. First, the soil moisture conditions of each plot were mapped using the relative saturation index of each cell (*I*_*ij*_), which represents the ratio of current and maximum soil volumetric water content prior to each event. Secondly, a threshold *Z* was set, and the value of each cell was replaced by *I*_*th*_:

4 $${I}_{th}=\{\begin{array}{cc}1 & if\,{I}_{ij} > Z\\ 0 & else\end{array}$$

**Figure 12:**
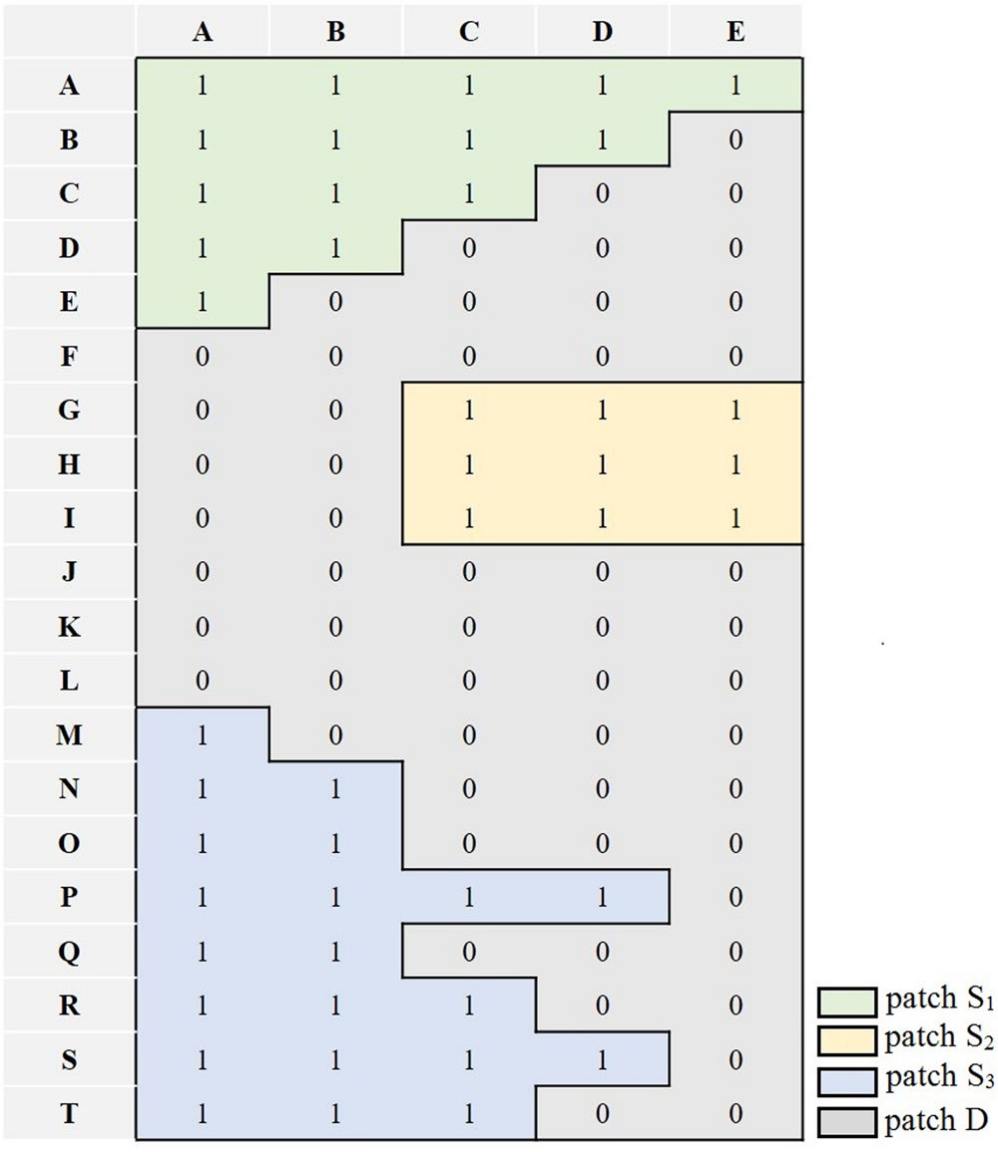
Example of an ICSL connectivity pattern.

The whole area was considered a spatial domain *G* while the subset *S*_*m*_ was the mth area made up of *I*_*th*_ = 1, and the connectivity function *τ(h)* is given by:

5 $$\tau (h)=P[{C}_{ij}\leftrightarrow ({C}_{ij}+h)|{C}_{ij}{\in }{S}_{m},({C}_{ij}+h){\in }{S}_{m}]$$

where *h* is the Euclidean lag distance, and *C*_*ij*_ and (*C*_*ij*_ + *h*) are two points in *G*, which can be considered as connected in the condition given by Eq. (4). Equation (5) represents the connected probability. The ICSL can be calculated as

6 $${\rm{ICSL}}={\int }_{0}^{\infty }(h)dh$$

that takes the average distance between each pair of connected points or cells^20^. An example is shown in Fig. 12 wherein the patches S_1_, S_2_, and S_3_ were the subset with *I*_*th*_ = 1, and the area *D* was the disconnected subset. *C*_*AA*_ and *C*_*CD*_ could be considered a connected pair.

Two more structure indices were proposed based on the following ICSL calculation processes: connected area proportion (CAP) and connectivity length (CL)

7 $$CAP=\frac{{A}_{C}}{{A}_{T}}\times 100 \% $$

where *A*_*C*_ is the connected area and *A*_*T*_ is the total area of the plot (100 m^2^), and CL is sum of the distance of each connected pair. Both indices were calculated for the threshold value of 0.3, and the values were analyzed relative to the SSFCs

The three thresholds of 0.3, 0.5, and 0.7 were set, and the ICSLs of each precipitation event under the thresholds were calculated to analyze the relationship between structural connectivity and SSFC. The ICSL and area were calculated using the bwlabel function from the Image Processing Toolbox in MATLAB (The Mathworks, Inc.). More details can be found in Western *et al*. (2001) and Ali and Roy (2010).
